# Monitoring of meteorological and hydrological droughts in the Vistula basin (Poland)

**DOI:** 10.1007/s10661-018-7058-8

**Published:** 2018-10-30

**Authors:** Katarzyna Kubiak-Wójcicka, Bogdan Bąk

**Affiliations:** 1 Department of Hydrology and Water Management, Faculty of Earth Sciences, Nicolaus Copernicus University, Lwowska 1, 87-100 Toruń, Poland; 2 Institute of Technology and Life Sciences, Aleja Hrabska 3, 05-090 Falenty, Poland

**Keywords:** Meteorological drought, Hydrological drought, Standardized Precipitation Index (SPI), Standardized Water-level Index (SWI), Standardized Runoff Index (SRI), Vistula basin, Poland

## Abstract

The article presents the course of meteorological droughts in Vistula subcatchments in years 1981–2010 and their influence on the occurrence of hydrological droughts. Using the Standardized Precipitation Index (SPI) as an indicator of meteorological drought on the one hand and the Standardized Water-level Index (SWI) and Standardized Runoff Index (SRI) as indicators of hydrological drought on the other, the mutual relationships between precipitation conditions and hydrological conditions were evaluated, as well as the relationships between the two drought types. Studies were conducted for three cumulative periods of these indices, of 12, 24, and 48 months. It was determined that meteorological droughts occurred earliest in the north-western and central part of the basin, and latest in areas lying above 300 m a.s.l. and in the south of Poland. Total duration, depending on the cumulative period, for SPI comprised from 38 to 41% of the analyzed period and for SWI (35–47%) and SRI (24–51%). The strongest relationships were identified in the central part of the Vistula (0.8 < *r* < 0.85), while the weakest relationships were recorded in the foothill region (*r* < 0.5). There were also indicated non-climate-related factors influencing those relationships (underground reservoirs, diverse Vistula water resource usage for municipal and industrial intake).

## Introduction

Drought is one of the most destructive natural phenomena, causing significant economic and social damage (Dobrovolski 2015). Contrary to other threats related to climate changes, such as floods, which are usually limited to small regions and often appear at relatively predictable time intervals, droughts are difficult to prognose and their duration and extent are hard to predict (Vicente-Serrano and López-Moreno 2005; Tsakiris et al. 2006; Mishra and Singh 2010). Drought is multidimensional. Some studies show that there are a number of bioclimatic comfort zones in which people feel comfortable. The assessment of drought as well as other climatic parameters is very important to determine ideal places for the thermal comfort of humans (Cetin 2015; Cetin et al. 2018a). Recent studies of drought stress, using remote sensing, show monitoring of drought stress and its impact on the assessment of the recreational potential of regions (Cetin et al. 2018b, c). Since the 1970s, there has been an increase in the frequency of meteorological droughts in many parts of Europe, also in Poland, which is attributed to climate change (Andréasson et al. 2004; Bordi et al. 2009; Kundzewicz 2009; Seftigen et al. 2013; Šebenik et al. 2017). Towards the end of the twentieth century, frequent occurrences of meteorological and hydrological droughts were identified in many parts of Poland, particularly in the years 1991–2000 (Somorowska 2009).

The problem of both drought types also affects the Vistula (Poland’s longest river) and its basin. This has been dealt with by many authors, although these works have been regional in nature (Kubiak-Wójcicka 2012; Bartczak et al. 2014; Meresa et al. 2016). The research by Kępińska-Kasprzak (2015) was a larger work on hydrological droughts of the main rivers of Poland. She analyzed these droughts for 1951–2000 and confirmed the impact of precipitation deficits on the occurrence of hydrological drought from 1 to 2.5 months after the occurrence of a shortage. The reaction time depended, among others, on air temperature increasing evaporation, particularly in the summer. In the case of heavy precipitation, surface waters reacted significantly more quickly than they did to deficits, and the drought usually ended in less than a month. Tokarczyk and Szalińska (2014) studied the course of meteorological and hydrological droughts in Poland’s mountain regions and lowlands. They confirmed that precipitation is more variable and hydrological conditions more dynamic in mountain rivers. Meanwhile, in lowlands, the tempo of changes in hydrological conditions is significantly slower; hence, the course and intensity of hydrological drought are more dependent on external factors. Generally, the reaction of catchments to a precipitation deficit is varied and depends primarily on the catchment’s physiographic features (permeability, topography, land use, and land cover), climatic conditions (precipitation and evaporation), and human activity (regulation of water levels, collection of water for community, and industrial purposes) (van Loon and Laaha 2015; Bąk and Kubiak-Wójcicka 2016).

The objective of the paper is to describe the Vistula basin in terms of the occurrence of meteorological droughts and hydrological droughts in the years 1981–2010. The statistics calculated for meteorological droughts made it possible to identify the regions of the Vistula basin most sensitive to such droughts and threshold parameter values for the onset of a drought (those drought parameters being: time of occurrence, drought magnitude, intensity, and sum of precipitation). Similar calculations were also made for the evaluation of hydrological drought.

The aforementioned parameters were identified based on the following drought indices: SPI (Standardized Precipitation Index) for meteorological drought and, for hydrological drought, SWI (Standardized Water-level Index), and SRI (Standardized Runoff Index). One key element of the research was the relations between meteorological and hydrological droughts. One practical result of the paper has been the development of a simple procedure for determining both the intensity of a meteorological drought based on ongoing sums of precipitation and the intensity of a hydrological drought on the Vistula. The knowledge gained may be useful in strategic water management planning in crisis situations (Barker et al. 2015), including on the level of the Vistula basin and on the regional level.

## Object of study and study area

The Vistula is the longest river in Poland, with a length of 1022 km. The area of the basin totals 194,000 km^2^, representing 54% of the area of Poland. Approximately 87% of the basin lies on Polish territory (GUS, Główny Urząd Statystyczny [Central Statistical Office]. Environment 2015). Vistula River is important to the landscape of Poland due to a variety of functions it plays in the region (Kubiak-Wójcicka et al. 2017).

**Fig. 1:**
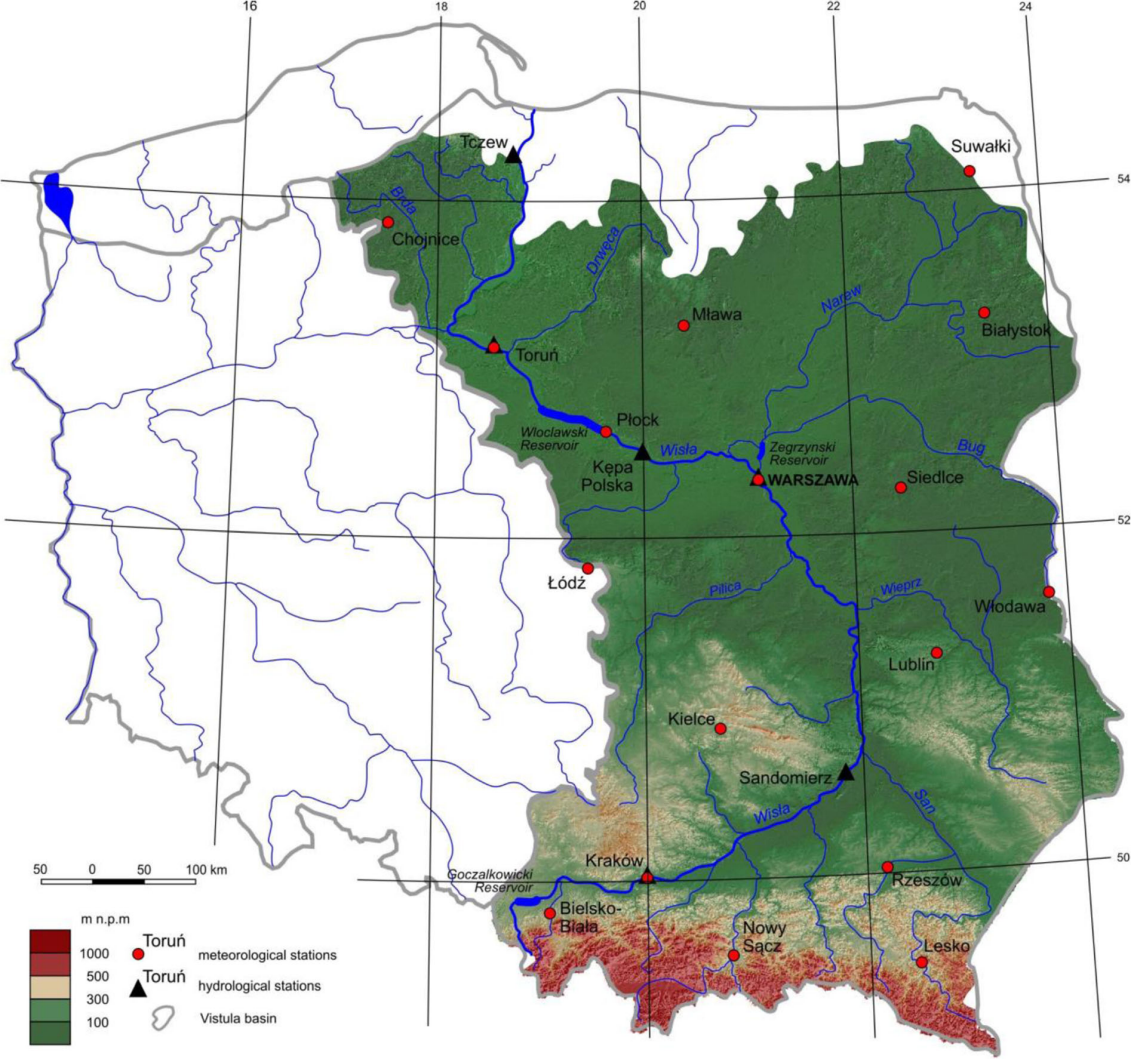
Vistula River basin

Average Vistula’s discharge for the period 1981–2010 measured on the gauge station in Tczew was 1027 m^3^ s^−1^. The majority of the Vistula basin is decidedly lowland in nature, with altitudes of predominantly up to 300 m a.s.l. (Fig. 1).

The Vistula has its sources on the slopes of Barania Góra (1106 m a.s.l.) and flows to the Baltic Sea, creating a delta in the Vistula Marshlands. The greatest water resources in the Vistula basin are found in the central Vistula basin, from the San to the mouth of the Narew (51%) and in the basin of the upper Vistula (approximately 38%). Its greatest tributaries are the following rivers: the San, the Narew and Bug, and the Pilica. There are two man-made reservoirs on the Vistula: the Goczałkowice reservoir is on the upper course of the river and the Włocławek reservoir is on the lower course. In addition to these, the Zegrzyński Lake reservoir on the lower course of the Narew has an influence on hydrological conditions in the middle section of the river (Kubiak-Wójcicka 2018). Along the Vistula, there are several subterranean water bodies, or Major Ground Water Basins (MGWB) which supply water for consumption and industrial purposes, particularly around the nation’s capital, Warsaw (Kleczkowski 1990).

## Climate and hydrological data

The analysis of meteorological and hydrological droughts used monthly sums of precipitation from 19 meteorological stations and average monthly water levels and runoffs from six hydrological stations. Data were taken from the Institute of Meteorology and Water Management-National Research Institute.

Studies of meteorological droughts in the Vistula basin and hydrological drought on the Vistula were conducted using the index method for the long-term period 1981–2010. These indices were calculated for cumulative periods of 12, 24, and 48 months the total precipitation, average runoff, or average water level.

It was assumed that each gauge station forms a closed subcatchment in which the selected precipitation measurement points are found. At the following locations of gauge stations along the Vistula’s course (Fig. 1), the number of precipitation stations in the subcatchment was, respectively: Kraków–3, Sandomierz–6, Warsaw Nadwilanówka–11, Kępa Polska–13, Toruń–17, and Tczew–19 (Table 1). SPI-48 calculation in January 1981 for subcatchment capped with gauge station in Toruń requires usage of monthly average precipitation values acquired from all 17 meteorological stations located in the subcatchment from 48 preceding months, i.e., since February 1977.

**Table 1:** Location of water gauges and gauges in the basin of the Vistula River; source: own study

Water gauge localization					
Kraków (69.2 km)	Sandomierz (268.4 km)	Warszawa (504.1 km)	Kępa Polska (606.5 km)	Toruń (734.7 km)	Tczew (908.6 km)
Gauge localization					
Bielsko Biała	Bielsko Biała	Bielsko Biała	Bielsko Biała	Bielsko Biała	Bielsko Biała
Kraków	Kraków	Kraków	Kraków	Kraków	Kraków
Nowy Sącz	Nowy Sącz	Nowy Sącz	Nowy Sącz	Nowy Sącz	Nowy Sącz
	Lesko	Lesko	Lesko	Lesko	Lesko
	Rzeszów	Rzeszów	Rzeszów	Rzeszów	Rzeszów
	Kielce	Lublin	Kielce	Kielce	Kielce
		Łódź	Lublin	Lublin	Lublin
		Włodawa	Łódź	Łódź	Łódź
		Siedlce	Włodawa	Włodawa	Włodawa
		Warszawa	Siedlce	Siedlce	Siedlce
			Warszawa	Warszawa	Warszawa
			Białystok	Olsztyn	Olsztyn
			Suwałki	Białystok	Białystok
				Płock	Płock
				Suwałki	Suwałki
				Mława	Mława
				Toruń	Toruń
					Bydgoszcz
					Chojnice

**Fig. 2:**
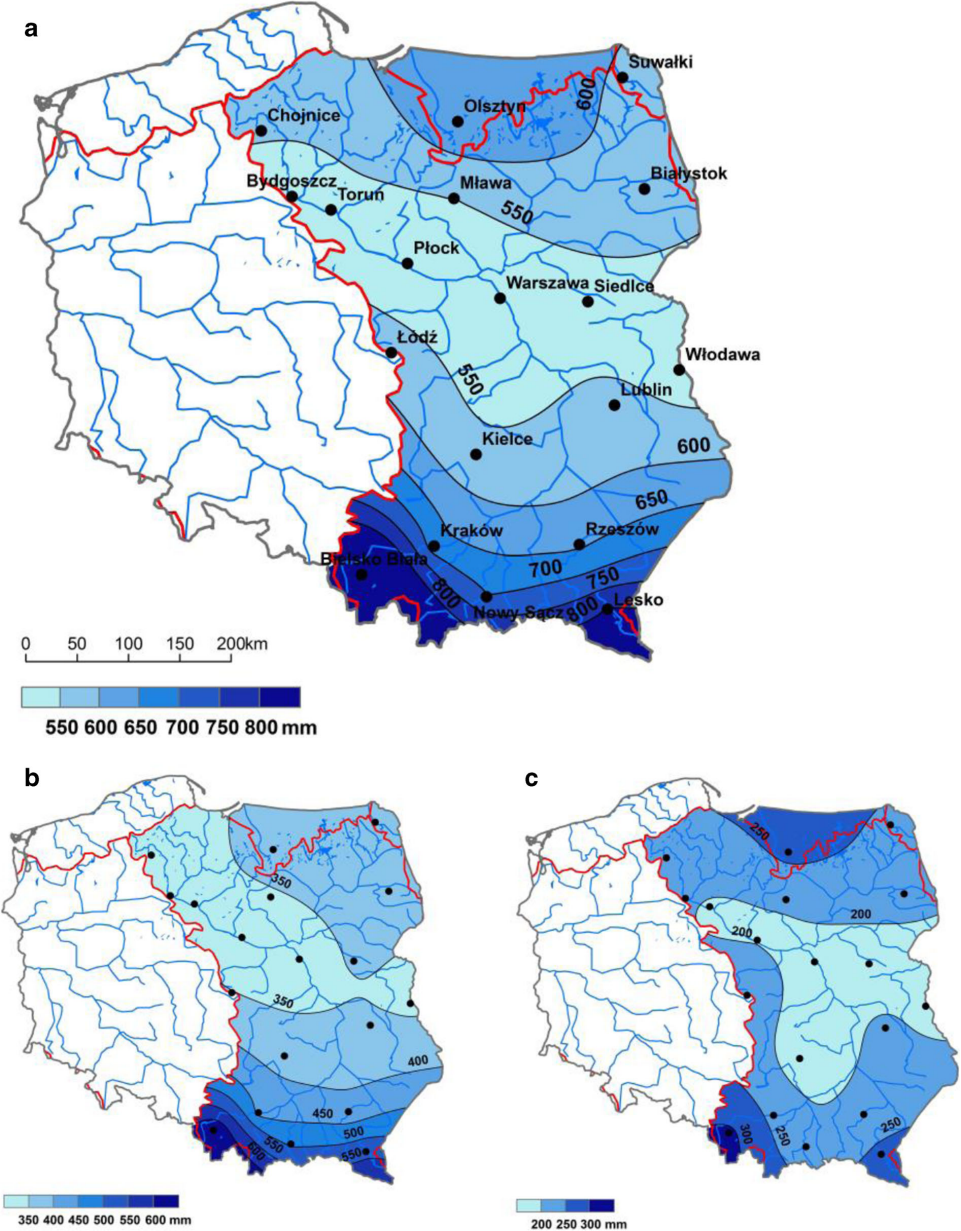
Spatial distributions of average sum of precipitation (mm) in the basin of the Vistula River (1981–2010): **a** year (Jan–Dec); **b** summer period (Apr–Sep); **c** winter period (Oct–Mar); source: own study

Analysis of precipitation in the Vistula basin showed an uneven distribution on the annual and seasonal scale (Fig. 2a–c). The distribution of annual precipitation in the Vistula basin can be distinguished into four zones. Firstly, the lowest sum of precipitation is found in the belt of lowlands in the center of the country and in the northern part of the Lublin Upland—550–600 mm—while, secondly, in the northern part of the basin, it is slightly greater, at 600–650 mm. The next two zones are areas above 300 m a.s.l., with 600–700 mm, and the south of the basin, with 700–1000 mm (Fig. 2a). The average sum of precipitation in the Vistula basin in 1981–2010 was 613 mm. Over the annual course, the driest month was February, with an average sum of precipitation of 29 mm in the basin, while the wettest were July (85 mm) and June (78 mm).

### Drought indices

The index method employed in the study allows the same calculation method to be used for the values of both drought types’ indices, in order to determine the same drought parameters and adopt a joint classification of drought intensity (Nalbantis and Tsakiris 2009; Bąk and Kubiak-Wójcicka 2016). Additionally, this method makes it possible to evaluate the relationships between precipitation and hydrological conditions in various time periods (months, seasons, years) based on the statistical correlations between index values (Folland et al. 2015).

The SPI index (McKee et al. 1995) used in the work has been widely used in recent years in literature (Nam et al. 2015; Hong et al. 2016; Santos et al. 2017; Zeleňáková et al. 2017). Since 2012, it has also been recommended by the World Meteorological Organization (WMO) for the operational monitoring of drought threats (World Meteorological Organization (WMO) 2012). Two indices are taken as hydrological drought indices: SWI, calculated on the basis of water level WL (Sahoo et al. 2015), and SRI, based on data for runoff *R* (Shukla and Wood 2008; Mishra and Nagarajan 2013; Li et al. 2016; Ljubenkov and Cindrić Kalin 2016; Zou et al. 2018). The values of the indices used are standardized deviations of precipitation, water levels, and runoff from median values for the long-term period. In the work, normal distribution fitting of homogeneous precipitation series was done using the transformer function *f(P)* = $\sqrt[3]{x}$ (Łabędzki 2017). In the case of water levels and runoff, the normalizing function adopted was the two-parameter logarithmic function, *ln* (Vicente-Serrano et al. 2012). Conformity of the distribution of the variable transformed by the function *f(P)* to the normal distribution was tested using the χ^*2*^ Pearson test. A positive conformity value allows the index value to be calculated by the formula:

1 $$ Z=\frac{f(X)-\widehat{\mu}}{\widehat{\delta}} $$

where: *Z* is the chosen index (*SPI, SWI*, *SRI*); *P*, *H*, *R* are the precipitation, water level, runoff; *f(X)* is the transformer of precipitation sums, water level, runoff; $\widehat{\mu}$ is the mean of normalized ***X***; $\widehat{\delta}$ is the standard deviation of normalized ***X.***

After McKee et al. (1995), it was assumed that, in a drought spell, all values of the indices SPI, SWI, and SRI are negative and, at the same time, in at least 1 month are less than or equal to − 1.0. The drought is broken if an index value goes above zero. The identified drought spells can most commonly be described with the adopted drought parameters: the drought parameters were as follows:

- *N* number of droughts.
- *D* duration [months].
- *Time* [%] percentage of total time of drought spells.
- *maxD* [months] duration of longest drought.
- *minD* [months] duration of shortest drought.
- ǀ*DM*ǀ total drought magnitude (sum of SPI values for all drought spells).
- *I [ǀDMǀ/D*] intensity.

For indices whose values fulfill the condition *X* < − 1.0 a joint, three-class evaluation of drought intensity was adopted: moderately dry (− 1.00 to − 1.49), severely dry (− 1.50 to − 1.99), and extremely dry (≤ 2.0).

The work uses series of cumulative sums of precipitation, water levels, and runoff as suggested in the literature for 12-, 24-, and 48-month periods, given as (*P*-12, *WL*-12, *R*-12, …, *P*-48, *WL*-48, *R*-48). Depending on the length of measurement series, the calculated drought index values were given the relevant designations (*SPI*-12, *SWI*-12, *SRI*-12, …, *SPI*-48, *SWI*-48, *SRI*-48). The relationships between the indices *SPI*-n and *SWI*-n and between *SPI*-n and *SRI*-n (*n* number of cumulative months) were evaluated using the correlation coefficients for successive months of the calendar year and for the entire year.

## Results

### Droughts in the years 1981–2010

#### Meteorological droughts

**Fig. 3:**
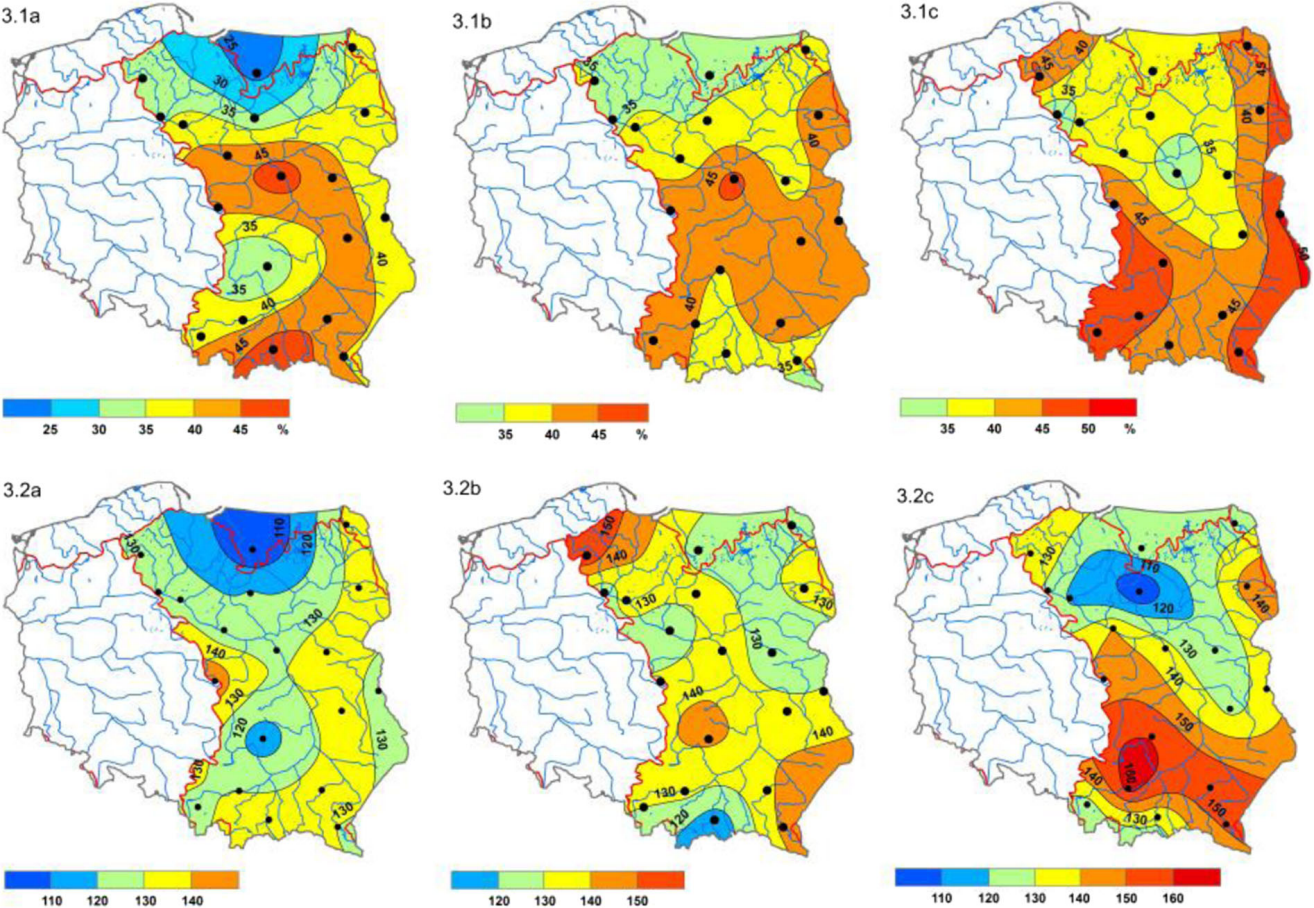
Spatial distributions of drought duration as percentage of total time (1): **1a** *SPI*-12; **1b** *SPI*-24; **1c** *SPI*-48 and total drought magnitude ǀDMǀ (2) in the basin of the Vistula River (1981–2010): **2a** *SPI*-12; **2b** *SPI*-24; **2c** *SPI*-48; source: own study

Based on the distribution of SPI-12 index values in the study period, an area of the northern part of the basin was distinguished in which the total duration of droughts was shortest, at 24% of the total multi-annual period, and in the central part of the basin and in the south two areas were identified where droughts lasted decidedly longer, i.e., 48% of the total study period. This difference in the total duration of droughts was 85 months. In the longer cumulative period of precipitation (*SPI*-24), droughts occurred most briefly in the northern and north-western part of the basin, i.e., for 32% of the total study period. In the center and east of the Vistula basin, this time was at least 5 to 7% longer, while in the middle of the basin, it was maximum 46%. For *SPI*-48, the shortest total drought duration occurred in the belt of lowlands running from the north-western extent of the basin to the center of the country and in the northern part of the Vistula basin, with a minimum of 31%. Significantly longer periods were identified in the south and eastern limits of the basin, with a maximum of 51%. The lowest total drought magnitudes (|DM|) for all variations of the *SPI* indicator were observed in the north-western and northern part of the Vistula basin. In eastern Poland, these were clearly greater, which may be explained by the longer durations of the phenomena (Fig. 3).

#### Hydrological droughts

Hydrological droughts on the Vistula, as determined by the *SWI*-n index, occurred several times during the long-term study period. Using a longer period for averaging water levels reduced the number of droughts over the long-term period. The total drought duration ranged from 33 to 46% for *SWI*-12, from 32 to 54% for *SWI*-24, and from 38 to 43% for *SWI*-48. The longest individual hydrological droughts as determined by the *SWI*-48 index lasted for around 150 months. Drought intensity in the long-term period ranged from − 0.8 to − 1.3 (depending on index). The strongest droughts were found in the center of Poland (Warsaw), and the weakest in Sandomierz. The reason for that may be huge water intake in Warsaw and, on the other hand, a large number of mountain tributaries feeding Vistula between Kraków and Sandomierz.

In most cases, the number of hydrological drought spells according to the *SRI*-n index was similar to the number of drought spells according to *SWI*-n. The greatest number of drought periods was recorded at Sandomierz (6; *SWI*-12). Over various time frames, cases of 1-month-long droughts were also recorded, in Warsaw, Kępa Polska, and Tczew. Also, for this index, total hydrological drought duration was variable, ranging from 23 to 48% between selected measurement points. The longest single drought lasted 151 months, and the shortest, 16. Drought intensity ranged from − 0.8 to − 1.3.

Averaged values of the statistics for meteorological and hydrological droughts are presented in Table 2.

**Table 2:** Statistics of mean parameter values for meteorological and hydrological droughts; source: own study

Index	*N*	*Time* [%]	*D* [months]	*maxD* [months]	*minD* [months]	Total *ǀDMǀ*	*I*
*SPI*-12	7	38	137	38	6	128	− 1.0
*SPI*-24	4	39	140	66	15	132	− 0.9
*SPI*-48	2	42	149	105	28	136	− 0.9
*SWI*-12	3	39	141	84	21	135	− 1.0
*SWI*-24	3	43	155	93	35	145	− 1.0
*SWI*-48	1	41	147	126	61	140	− 1.0
*SRI-12*	4	39	139	73	20	118	− 0.8
*SRI-24*	3	47	168	84	31	134	− 0.8
*SRI-48*	1	34	122	112	47	110	− 0.9

### The course of meteorological and hydrological droughts and their interrelations

**Fig. 4:**
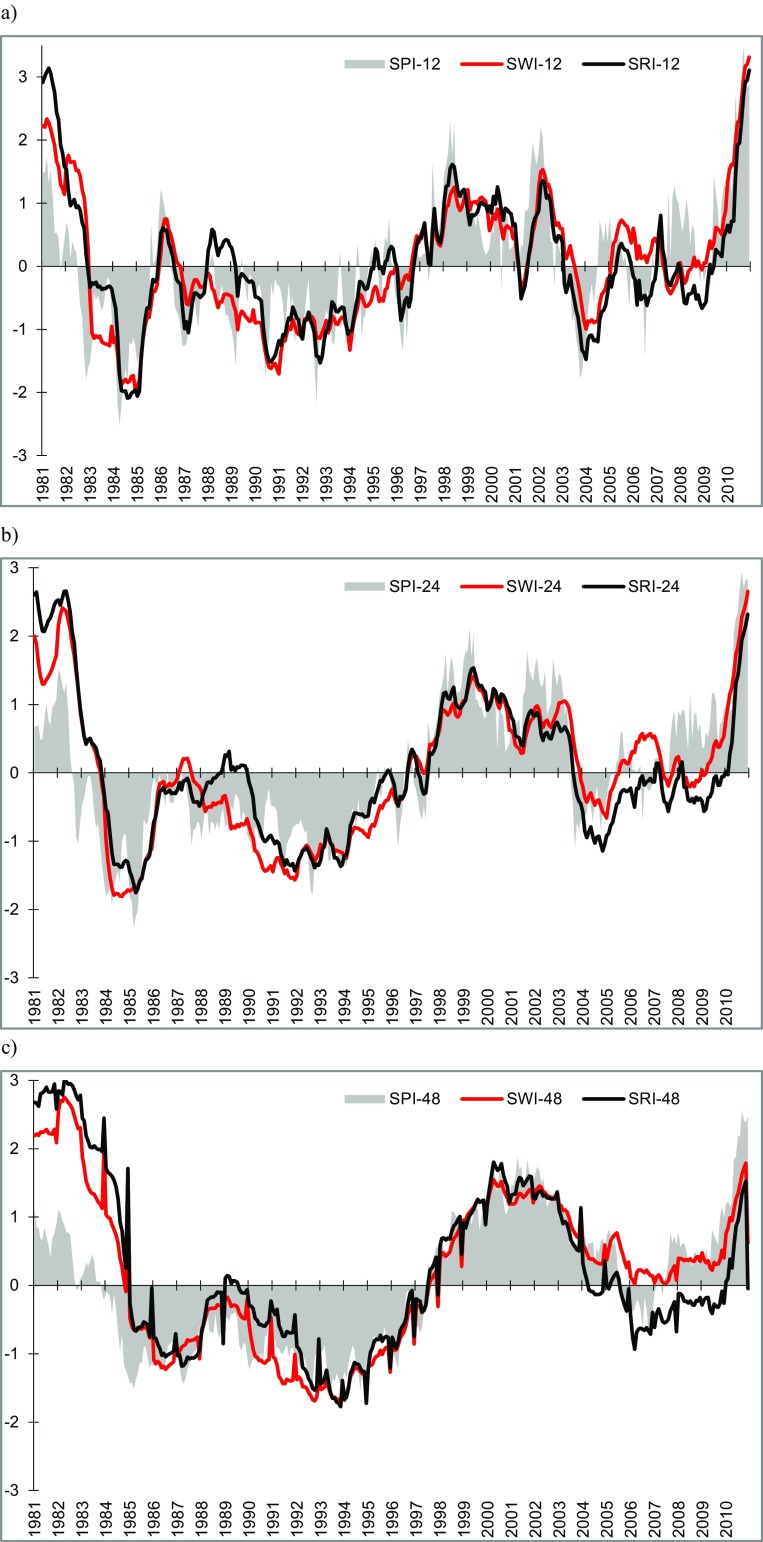
Course of SPI, SWI, and SRI at the Kępa Polska hydrological gauge in the accumulation periods of **a** 12, **b** 24, and **c** 48 months

An example of parallel courses of meteorological and hydrological droughts in subcatchments is shown for the measurement station at Kępa Polska (Fig. 4). The choice of this location was dictated by the fact that the Vistula’s channel over this section is unmanaged and natural; there is no influence of any water reservoir and it is located beyond the reach of MGWB underground reservoirs. This station covers 87% of the total area of the Vistula basin.

This example shows that meteorological and hydrological drought spells significantly coincided with one another over the long-term period. The 12-month cumulative period for precipitation and averaged water levels and runoff had a greater variability of drought indices, which caused the total duration of simultaneous occurrence of both drought types to be shortest for this cumulative period. In the longer periods (24 and 48 months), the duration of simultaneous occurrence was longer.

The extent of correlation between precipitation and hydrological conditions at all stations was established by calculating the correlation coefficients *r* of *SPI*-n to *SWI*-n and of *SPI*-n to SRI-n. This study was done in two variants: for the relationships of all index values in successive months and on the annual scale and for the relationships in the period limited only to meteorological drought spells and both kinds of hydrological drought.

The data presented in Table 3 show the dependence of hydrological conditions on precipitation to be highly variable. The minimum value of *r* was 0.28, and the maximum was 0.92. It is important to note here that in most cases, the correlations were high (0.7 < *r* < 0.9). Such relationships are exemplified at, inter alia, Sandomierz: *SPI*-12 to *SWI*-12 (*r* = 0.72), *SPI*-12 to *SRI*-12 (*r* = 0.88), *SPI-24* to *SRI*-24 (*r* = 0.89), and in Tczew: *SPI*-12 to *SWI*-12 (*r* = 0.77), *SPI*-24 to *SWI*-24 (*r* = 0.83), *SPI*-48 to *SWI*-48 (*r* = 0.86). Only in Kraków, in the upper course of the Vistula, were values of *r* smaller for all variants. Here, the greatest correlation was found between *SPI*-12 and *SWI*-12 (*r* = 0.63).

**Table 3:** Correlation coefficients: *SPI*-n to *SWI*-n and *SPI*-n to *SRI*-n (*n* = 12, 24, 24); source: own study

	I	II	III	IV	V	VI	VII	VIII	IX	X	XI	XII	I-XII
Kraków													
SPI-12 vs. SWI-12	0.66	0.63	0.65	0.63	0.58	0.59	0.62	0.57	0.61	0.65	0.65	0.67	0.63
SPI-24 vs. SWI-24	0.55	0.57	0.59	0.57	0.52	0.54	0.57	0.49	0.52	0.53	0.54	0.56	0.54
SPI-48 vs. SWI-48	0.42	0.42	0.43	0.41	0.38	0.40	0.46	0.42	0.39	0.40	0.40	0.28	0.40
Sandomierz													
SPI-12 vs. SWI-12	0.77	0.78	0.76	0.73	0.71	0.70	0.66	0.66	0.68	0.68	0.72	0.79	0.72
SPI-12 vs. SRI-12	0.91	0.91	0.91	0.89	0.90	0.88	0.84	0.84	0.86	0.86	0.87	0.92	0.88
SPI-24 vs. SWI-24	0.63	0.66	0.67	0.64	0.64	0.66	0.63	0.62	0.62	0.62	0.65	0.69	0.64
SPI-24 vs. SRI-24	0.88	0.88	0.90	0.89	0.90	0.90	0.87	0.86	0.87	0.88	0.89	0.91	0.89
SPI-48 vs. SWI-48	0.53	0.53	0.53	0.50	0.49	0.51	0.53	0.49	0.47	0.48	0.49	0.29	0.49
SPI-48 vs. SRI-48	0.87	0.87	0.88	0.87	0.87	0.88	0.88	0.87	0.86	0.87	0.87	0.66	0.86
Warsaw													
SPI-12 vs. SWI-12	0.54	0.54	0.57	0.52	0.53	0.54	0.54	0.53	0.46	0.49	0.47	0.54	0.52
SPI-12 vs. SRI-12	0.86	0.86	0.86	0.80	0.83	0.80	0.74	0.82	0.81	0.79	0.81	0.86	0.82
SPI-24 vs. SWI-24	0.56	0.56	0.59	0.55	0.56	0.54	0.55	0.54	0.51	0.52	0.50	0.54	0.54
SPI-24 vs. SRI-24	0.82	0.83	0.85	0.85	0.85	0.85	0.80	0.83	0.81	0.81	0.84	0.86	0.83
SPI-48 vs. SWI-48	0.61	0.61	0.62	0.60	0.61	0.60	0.61	0.63	0.61	0.62	0.59	0.64	0.61
SPI-48 vs. SRI-48	0.84	0.84	0.85	0.84	0.83	0.85	0.85	0.81	0.80	0.81	0.82	0.58	0.81
Kępa Polska													
SPI-12 vs. SWI-12	0.78	0.79	0.81	0.78	0.82	0.77	0.68	0.73	0.70	0.66	0.68	0.78	0.75
SPI-12 vs. SRI-12	0.68	0.70	0.74	0.71	0.75	0.70	0.59	0.64	0.62	0.56	0.59	0.68	0.66
SPI-24 vs. SWI-24	0.83	0.87	0.89	0.86	0.90	0.86	0.78	0.79	0.75	0.72	0.75	0.83	0.82
SPI-24 vs. SRI-24	0.71	0.74	0.77	0.79	0.81	0.80	0.73	0.73	0.70	0.68	0.71	0.76	0.74
SPI-48 vs. SWI-48	0.86	0.87	0.87	0.85	0.87	0.87	0.84	0.82	0.79	0.78	0.80	0.61	0.82
SPI-48 vs. SRI-48	0.72	0.73	0.75	0.73	0.74	0.75	0.74	0.69	0.66	0.66	0.68	0.43	0.69
Toruń													
SPI-12 vs. SWI-12	0.60	0.62	0.65	0.61	0.64	0.58	0.47	0.50	0.47	0.44	0.49	0.58	0.55
SPI-12 vs. SRI-12	0.74	0.77	0.80	0.77	0.82	0.75	0.66	0.70	0.64	0.61	0.63	0.73	0.72
SPI-24 vs. SWI-24	0.52	0.55	0.57	0.57	0.57	0.55	0.48	0.45	0.42	0.40	0.45	0.49	0.50
SPI-24 vs. SRI-24	0.70	0.73	0.76	0.78	0.80	0.78	0.71	0.70	0.67	0.64	0.67	0.72	0.72
SPI-48 vs. SWI-48	0.48	0.49	0.49	0.47	0.45	0.46	0.46	0.39	0.34	0.35	0.37	0.16	0.41
SPI-48 vs. SRI-48	0.71	0.72	0.74	0.73	0.73	0.74	0.71	0.67	0.63	0.63	0.64	0.38	0.67
Tczew													
SPI-12 vs. SWI-12	0.81	0.83	0.86	0.83	0.87	0.79	0.73	0.75	0.67	0.67	0.69	0.79	0.77
SPI-12 vs. SRI-12	0.70	0.73	0.78	0.75	0.79	0.71	0.60	0.64	0.59	0.56	0.58	0.68	0.68
SPI-24 vs. SWI-24	0.83	0.86	0.88	0.88	0.90	0.86	0.83	0.82	0.76	0.75	0.78	0.82	0.83
SPI-24 vs. SRI-24	0.65	0.68	0.72	0.73	0.76	0.73	0.66	0.64	0.61	0.58	0.61	0.66	0.67
SPI-48 vs. SWI-48	0.90	0.91	0.91	0.91	0.92	0.92	0.89	0.87	0.83	0.84	0.85	0.64	0.86
SPI-48 vs. SRI-48	0.66	0.68	0.69	0.68	0.69	0.69	0.66	0.62	0.58	0.57	0.58	0.34	0.62

Such diverse results were also obtained in studies of the relationship between droughts and their time of occurrence. On all gauge stations, the values of *r* in the periods of parallel appearance of both kinds of droughts were enclosed in the range of 0.62 < *r* < 0.70. These can be broken down into three groups: very weak or no relationship (*r* < 0.5), moderate relationship (0.5 < *r* < 0.6), and good relationship (*r* > 0.6) (Bachmair et al. 2015).

### Threshold of drought and nomograms

The joint classification of drought spells adopted in the work allowed threshold values to be determined for the analyzed parameters of classes of drought, using an inversion of formula 1. An example of maps of the distribution of threshold precipitation values for the onset of drought (SPI = − 1.0) in the adopted cumulative periods of precipitation is presented in Fig. 4.

Transforming the formula [1] to the form of [2], threshold precipitation values *P*-n (*n* = 12. 24. 48) may be calculated in each precipitation gauge in the Vistula basin, below which moderate (*SPI*-*n* = − 1.0), severe (*SPI*-*n* = − 1.5), and extreme (*SPI*-*n* = − 2.0) droughts begin:

2 $$ P={\left( SPI\cdot \mu +\delta \right)}^3 $$

where:

*P*: threshold of drought beginning (mm),
*μ*: mean of normalized precipitation,
*δ*: standard deviation of normalized precipitation; source: own study.

The results achieved in the study show that meteorological drought in the Vistula basin did not appear at the same time as hydrological drought. It was ascertained that at each meteorological station, the precipitation threshold value for a drought was different. In the south of Poland (mountain region), the thresholds were the highest. For example, meteorological drought in Bielsko Biała appeared when 12-month precipitation sum was less than 814 mm while in Nowy Sącz 613 mm. In the highlands, the thresholds were much lower, e.g., in Kraków 560 mm and in Lublin 491 mm. The lowest threshold values were found in lowlands of central Poland: in Bydgoszcz 405 mm and in Toruń 424 mm. Threshold values calculated for longer intervals were proportionally greater than the values for 12-month periods. For example, in Warsaw, the threshold for meteorological drought SPI-12 was 574 mm, SPI-24 1178 mm, and SPI-48 2043 mm.

Average precipitation of all meteorological stations in a given subcatchment was assumed the threshold precipitation values. In the 12-month period, threshold precipitation levels in particular subcatchments were Kraków 673 mm, Sandomierz 632, Warsaw 574, Kępa Polska 572, Toruń 560, Tczew 555. Similar calculation method was applied to hydrological gauge stations. In the same period, the threshold water level in Warsaw was 2285 cm and the threshold discharge 5229 m^3^ s^−1^. In 24-month period, the values were, respectively, 4643 cm and 9680 m^3^ s^−1^, and in 48-month period: 9466 cm and 19,554 m^3^ s^−1^.

**Fig. 5:**
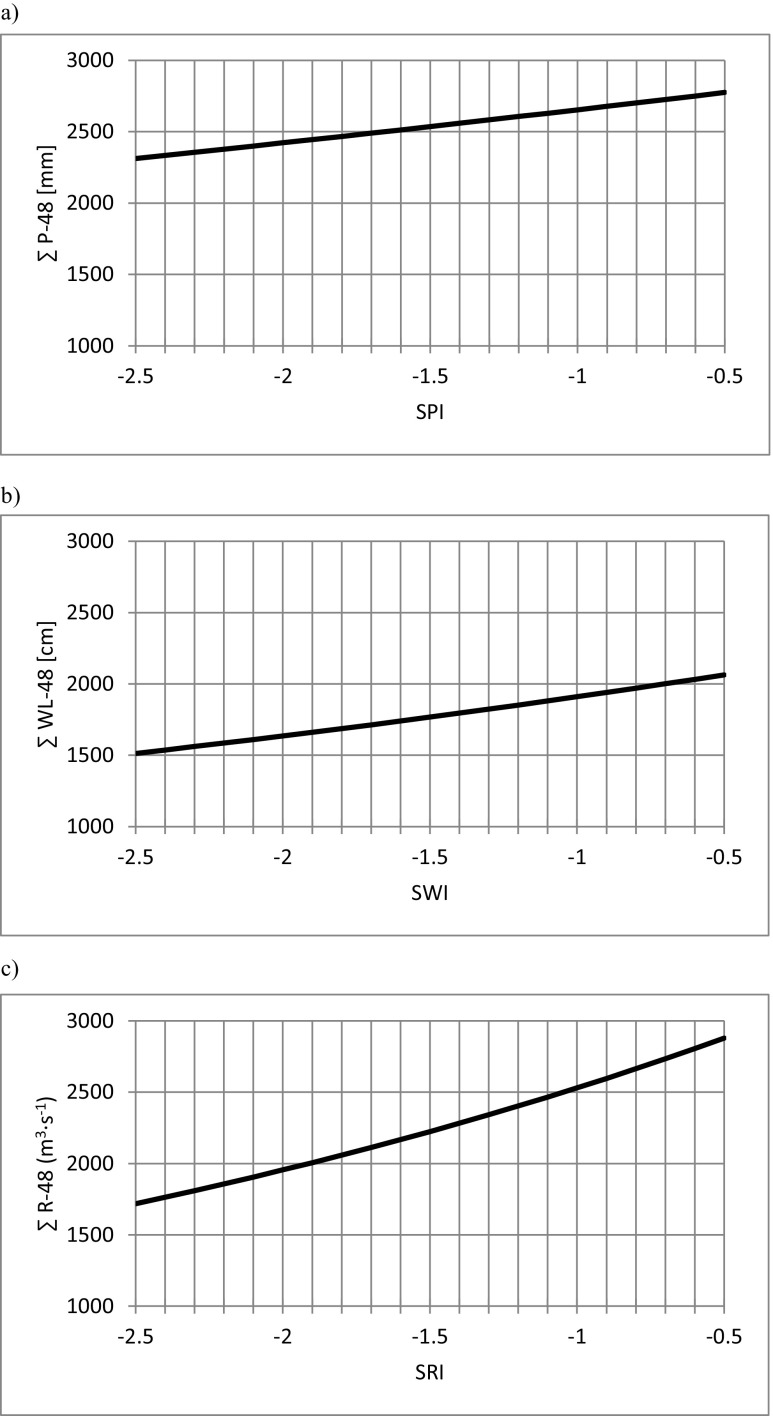
Nomograms to determine parameters of droughts: **a** meteorological (P → SPI); **b** hydrological (WL → SWL); **c** hydrological (R → SRI) for Sandomierz hydrological gauge; source: own study

Nomogram usage is simple. On the vertical axis, one marks either cumulated precipitation sum or water level or discharge, respectively. Casting the value on the linear regression chart allows determination of an appropriate index’ value on the horizontal axis (Fig. 5).

In the subcatchments, in which relationship between both types of drought were strong, there is a linear relation between the values of cumulated precipitation sums, water levels and discharges, and the corresponding index values. Usage of the nomograms facilitates monitoring of both drought type intensity. Depending on subcatchment location, the threshold values of cumulated precipitation sums, water levels, and discharges are different and depend on the assumed time scale. The examples prepared for the subcatchment in Sandomierz for 48-month period have been presented in Fig. 5.

## Discussion

The paper aims to identify the occurrence of hydrological drought in relation to meteorological droughts in Poland in the period 1981–2010. The analyzed problem is part of the latest research trend towards developing different approaches to the assessment and identification of drought. For the first time, the problem of drought in Poland was considered over a larger spatial scale, i.e., the Vistula River Basin, using the index method. The index method which was used includes identification of meteorological and hydrological droughts.

Data analysis showed that the lowest sums of precipitation and most frequent meteorological droughts occurred in central and central-eastern Poland. Similar results are given by Doroszewski et al. (2014), who analyzed the distribution of meteorological droughts in Poland for 1961–2010. Asymmetric precipitation distribution over Vistula basin has been presented on Fig. 1b–d. It may be assumed that the Vistula is a natural border between humid air flowing from the Atlantic Ocean and dry, continental air flowing from Russia. That fact is confirmed by precipitation distribution studies in Central Europe and Poland which have been conducted in 2nd half of 20th century and at the beginning of 21st century (Marosz et al. 2011). The extensive meteorological droughts in the Vistula basin in the years 1983, 2000, 2003, and 2006, identified in the work, formed part of a cross-border drought area extending beyond the borders of Poland (Rimkus et al. 2012, 2013; Vido et al. 2015).

The existence of extensive droughts across Europe in the years 1950–2012 has been confirmed by the research of Spinoni et al. (2015), who analyzed the occurrence of droughts in Europe based on the *SPI*-12 index. They divided Europe into 13 regions, with Poland classified into Eastern Europe (along with Bulgaria, Czech Republic, Hungary, Romania, Slovak Republic). They determined that eight long-term droughts occurred in their study period, including six droughts in the years 1991–2010. In the neighboring Ex-USSR region (Belarus, Moldova, Ukraine), they identified six droughts, and four in the Baltic Republics (Estonia, Latvia, Lithuania). The results of the current paper showed variable precipitation in the Vistula basin, typical of the distribution of precipitation in neighboring regions. It would appear that, due to the similarity of rainfall distribution and occurrence of droughts, the eastern part of the Vistula basin may be analyzed together with the Ex-USSR region and the north-eastern part together with the Baltic Republics. A significant part of the Vistula basin is made up of right tributaries in eastern Poland, constituting half of the water resources of the river basin. In turn, hydrological droughts in the upper course of the Vistula, which covers the southern part of the basin, are dependent on the extent of precipitation in the Carpathians (Fal 2007; Vido et al. 2015).

Analysis herein of the distribution of meteorological and hydrological droughts identified the following instances:

1. no meteorological or hydrological droughts (e.g., in 1981, 1999, and 2001),
2. meteorological droughts (Toruń: June 1984–Dec 1990),
3. hydrological drought without meteorological drought (Tczew: Sep 2003–Mar 2010),
4. concurrent meteorological and hydrological droughts (Warszawa: Jan 1985–Dec 1992; Kępa Polska: Jan 1985–June 1997). The above cases were also observed in other regions of Europe at the turn of the twenty-first century (Blauhut et al. 2015).

This work analyzed the course of hydrological droughts over the annual course because ice phenomena appear on the Vistula River in the winter (although sporadically in recent years and never along the whole length of the river) (Cyberski et al. 2006; Pawłowski 2009). Cases of river beds freezing and long-term ice phenomena, i.e., lasting from late autumn to the beginning of spring, were recorded in the southern and eastern part of the Vistula basin. The problem of frozen rivers was also noted by Rimkus et al. (2013), who studied the relationship between meteorological and hydrological droughts in the Neman river basin (Lithuania, Latvia). Those authors suggested in such cases investigating dependences during periods of free water flow only, i.e., in summer and autumn. In the case of the Vistula, the minimum average monthly water levels were most frequently observed in those very periods and were one third lower than the average monthly values in the long-term study period. Meanwhile, minimum average monthly runoff in summer and autumn was half the average for the long-term study period.

In analyzing the interrelations of both drought types, it is important to take into account non-climatic factors which may locally influence the Vistula’s hydrological regime (Tokarczyk and Szalińska 2014; Bąk and Kubiak-Wójcicka 2017). According to van Loon and Laaha (2015), there are more than 30 factors in a catchment that can influence the occurrence of hydrological drought. Examples of such influences can also be found in the Vistula basin. These include the influence that man-made reservoirs, such as the Goczałkowice, or, to a lesser degree, the Włocławek reservoir and the other reservoirs on the tributaries of the Vistula have on the Vistula’s hydrological regime. In addition, a lot of water is extracted at Warsaw for consumption and industrial purposes. Around the analyzed water gauges and their surrounding areas, there are groundwater reservoirs of various capacities which significantly contribute to the Vistula channel during precipitation deficits.

Dramatic changes in the interactions between external factors in the Vistula basin, and also the appearance of extreme climatic conditions in the Vistula basin, may have an influence on the appearance or disappearance of a hydrological drought, regardless of the existence of a meteorological drought. This is most frequently observed over short periods of 1 to 3 months. The large area of the Vistula basin and the role of groundwaters in the feeding of the river channel determine the significant hydrological inertia of the river. For this reason, the long cumulative precipitation periods (12, 24, and 48 months) which were adopted cause the relations between meteorological and hydrological droughts to be large (*r* > 0.7). The small correlation coefficients between droughts identified in the Neman river basin may result from the adoption of too short a cumulative precipitation period. Shorter cumulative periods (under 12 months) are most commonly used in small catchments in a dry and Mediterranean climate (Loukas and Vasiliades 2004; Haslinger et al. 2014). In many cases, the *r* values obtained were higher than the assumed threshold values, and these relationships can be considered very strong (Bachmair et al. 2015).

The practical dimension of this work resides in the attached nomograms, which allow values of a meteorological and hydrological drought index to be determined based on the sum of precipitation over the given periods of time. The nomograms proposed by the authors are new and were inspired by the work of Bąk (2006), who used similar nomograms, based on current precipitation, to determine the relationship between precipitation and a meteorological drought, and between meteorological drought and agricultural drought. In turn, Al-Faraj et al. (2014) used nomograms to determine drought index values: RDIst (Standardized Reconnaissance Drought Index) and SDI (Streamflow Drought Index) depending on changes in precipitation and potential evaporation. In the case of the Vistula basin, the proposed nomograms are useful in, for example, predicting the intensity of particular types of drought. Such knowledge may be valuable for, among others, decision-making water management bodies.

Vistula River and its basin is an important source of water for the community and the economy. Water resources in Poland belong to the least in Europe (Gutry-Korycka et al. 2014). Hence, assessment of meteorological and hydrological drought occurrence threat is an important issue. The proposed nomograms may be utilized for purposes of that threat monitoring.

The relationship between meteorological and hydrological types of drought has not been assessed with use of the standardized drought indices in the Vistula basin so far. This relationship turned out to be stronger in the regions with large water intake from the river. Such conclusions could be found thanks to the new approach to the standardized index method, i.e., assessing the relationships in particular subcatchments.

## Conclusions

The main objectives of the work were to describe the Vistula basin in terms of the occurrence of meteorological and hydrological droughts observed in various sections of the Vistula river and to identify their mutual relations. It was assumed that the main factor causing hydrological drought is the occurrence of a long period of low precipitation, or, particularly, of no precipitation. Large rivers do not always react to short-term meteorological droughts due to the hydrological inertia of the basin. Only under the influence of several months of low precipitation, or lack thereof, do signs of hydrological drought appear.

Analysis of meteorological and hydrological droughts was carried out based on the values of the standardized indices (SPI, SWI, and SRI) in the period 1981–2010. The index method which was used allowed the joint method to be used in determining the dates of occurrence of both droughts in the multi-year study period and the same drought parameters to be calculated. This task was performed over three time scales of 12, 24, and 48 months.

The literature and the authors’ own research have shown that in many cases, where meteorological droughts were observed, the Vistula river basin was part of a much larger drought zone covering several to a-dozen-or-more countries in Europe. The spatial distribution of meteorological droughts was similar to the distribution of precipitation; they occurred earliest in the lowlands, and latest in the Carpathian Foothills. It is worth remembering here that the threshold values for sum of precipitation differed significantly across the entire river basin.

The effects of reduced precipitation in the Vistula basin were observed in changes in water levels and runoff at the measurement stations. During summer and early-autumn meteorological droughts, water levels and runoff were clearly decreasing. The high correlation coefficient values of SPI to SWI and of SPI to SRI in successive months and on the yearly scale indicate the influence of precipitation on the adopted hydrological parameters. Relationships limited to periods of concurrent occurrence of both types of drought were weaker, and in some cases, especially SPI to SWI, were very weak or exhibited no relationship.

The obtained research material has significantly broadened the current understanding of the threat of meteorological drought in the Vistula basin and its potential effects in the form of hydrological drought on the Vistula River. The relationships identified between droughts were not always unambiguous, and the probable cause was the influence of external factors.
